# Ribosomal DNA in diploid and polyploid *Setaria* (Poaceae) species: number and distribution

**DOI:** 10.3897/CompCytogen.v9i4.5456

**Published:** 2015-10-07

**Authors:** Thaís Furtado Nani, Gisele Cenzi, Daniele Lais Pereira, Lisete Chamma Davide, Vânia Helena Techio

**Affiliations:** 1 Federal University of Lavras, Department of Biology, Zip Code 37.200-000, Lavras, Minas Gerais State, Brazil

**Keywords:** Karyotype, FISH, forage species, chromosomal rearrangements

## Abstract

*Setaria* Beauvois, 1812 is a genus of economically important forage species, including *Setaria italica* (Linnaeus, 1753) Beauvois, 1812 and *Setaria viridis* (Linnaeus, 1753) Beauvois, 1812, closely related species and considered as model systems for studies of C4 plants. However, complications and uncertainties related to taxonomy of other species of the genus are frequent due to the existence of numerous synonyms for the same species or multiple species with the same name, and overlapping of morphological characteristics. Cytogenetic studies in *Setaria* can be useful for taxonomic and evolutionary studies as well as for applications in breeding. Thus, this study is aimed at locating 45S and 5S rDNA sites through fluorescent *in situ* hybridization (FISH) in *Setaria italica*, *Setaria viridis* and *Setaria sphacelata* (Schumacher, 1827) Stapf, Hubbard, Moss, 1929 cultivars (cvs.) Narok and Nandi. *Setaria italica* and *Setaria viridis* have 18 chromosomes with karyotype formulas 6m + 3sm and 9m, respectively. The location of 45S and 5S rDNA for these species was in different chromosome pairs among the evaluated species. *Setaria viridis* presented a more symmetrical karyotype, strengthening the ancestral relationship with *Setaria italica*. *Setaria sphacelata* cvs. Narok and Nandi have 36 chromosomes, and karyotype formulas 11m+7sm and 16m+2sm, respectively. The 45S rDNA signals for both cultivars were also observed in distinct chromosome pairs; however chromosomes bearing 5S rDNA are conserved. Karyotypic variations found among the studied species are evidence of chromosomal rearrangements.

fluorescent *in situ* hybridization

## Introduction

*Setaria* Beauvois, 1812 is a genus of the family Poaceae Barnhart including 125 cultivated, wild or weed species distributed in the warmer temperate regions worldwide (Gressel 2005). This genus includes Foxtail millet (*Setaria italica* (Linnaeus, 1753) Beauvois, 1812) and its wild ancestor Green foxtail (*Setaria viridis* Linnaeus, 1753) Beauvois, 1812), which have been considered as model systems for studies of C4 plants, stress biology and biofuel production (Muthamilarasan and Prasad 2015). *Setaria italica* is important for production of forage (McCartneyet al. 2009) and cereal for human consumption, especially in China, India, Japan, Russia and Nepal (Wanous 1990). *Setaria sphacelata* (Schumacher, 1827) Stapf, Hubbard, Moss, 1929, known as Golden bristle grass, is also important for its use in the formation of grasslands (Jones and Ford 1972).

Cytogenetic descriptions demonstrate that over 82% of the genus consists of polyploid species (Caponio and Pensiero 2002), with basic number x=9 (Darlington and Wylie 1955). Studies performed by Benabdelmouna et al. (2001) and Zhao et al. (2013) show that six distinct genomes are part of the evolutionary history of the genus. *Setaria italica* and *Setaria viridis* have 2n=18 chromosomes belonging to the genome A (Benabdelmouna et al. 2001) and details of chromosome morphology were presented by Croullebois and Siljak-Yakovlev (1989) and Benabdelmouna et al. (2001). The chromosome number of *Setaria sphacelata* varies from 2n=18 to 2n=90 (Hacker and Jones 1969), however, there are no descriptions on the chromosomal morphology.

In the genus *Setaria*, complications and uncertainties related to taxonomy are common due to the existence of numerous synonyms for the same species or multiple species with the same name, and overlapping of morphological characteristics. (Missouri Botanical Garden 2014). Taxonomic divergences represent a problem for breeders and for the exchange and conservation of genetic resources, because many plants can be wrongly incorporated in germplasm collections or incorrectly used in crosses.

Studies using molecular markers (Wang et al. 1998), isozymes (Kawase and Sakamoto 1984) and comparisons between genomes through GISH technique (Genomic *In Situ* Hybridization) (Benandelmouna et al. 2001) have been made on *Setaria italica* and *Setaria viridis*, and the results increasingly confirm the proximity between these species. The only justification for maintaining two separate taxa is the history of domestication of these species, which allowed them to become seemingly distinct groups in morphology, growth and agronomic use (Benandelmouna et al. 2001).

The chromosome number is an important datum in cytotaxonomic studies, and when combined with size, morphology, karyotype symmetry, banding patterns and satellite DNA position in the chromosome, enables a better understanding of karyotype evolution between species (Greilhuber 1995). The fluorescence in situ hybridization (FISH) technique allows the identification of specific regions in the physical mapping of chromosomes, and enables more accurate karyotypic comparisons among species, cultivars and populations (Guerra 2008), especially in the case of species with small chromosomes, since specific signals facilitate differentiation of chromosomes (Galasso et al. 1995).

rDNA probes are widely used in cytogenetic studies as they can contribute with information about homology between chromosomal segments, especially among related species (Britton-Davidian et al. 1995). Studies on rDNA of *Setaria* species began with Benabdelmouna et al. (2001), who identified signals of 18S-5, 8S-25S and 5S rDNA in chromosomes of *Setaria adhaerens* (Forsskål, 1775) Chiovenda, 1919, *Setaria faberi* Herrmann, 1910, *Setaria italica*, *Setaria verticillata* (Linnaeus, 1762) Beauvois, 1812 and *Setaria viridis*. However, the chromosome pairs with ribosomal DNA signals were not identified, and there are no reports on the location of 45S and 5S rDNA in *Setaria sphacelata*. In addition, this information was not used in cytotaxonomic and evolutionary approaches.

More detailed cytogenetic comparisons between diploid and polyploid species are important to increase the knowledge and understanding of the relationship between species of the genus. In this context, this study analyzed the karyotype using the location of 45S and 5S rDNA in the chromosomes of *Setaria sphacelata* (cultivars Nandi and Narok), *Setaria italica* and *Setaria viridis*. The results will contribute to understand the chromosomal/genomic organization of this genus and can produce useful information for taxonomic and breeding studies.

## Material and methods

We evaluated diploid genotypes of *Setaria italica* variety yugul and *Setaria viridis* variety A10.1, from roots collected in Missouri - United States, and tetraploid genotypes of *Setaria sphacelata*, cultivars Nandy and Narok, from the germplasm bank of forage plants of the Department of Animal Science, Federal University of Lavras, Minas Gerais State, Brazil.

Root tips were collected and pretreated with 3 mM 8-hydroxyquinoline at 0-4 °C for 4 hours, fixed in Carnoy for 20 minutes and subsequently stored in 70% ethanol at -20 °C until use. For the preparation of the slides by the flame-drying technique, the roots were previously digested in a solution of 4% cellulase and 2% pectinase at 37 °C for about 1 hour and 20 minutes.

Mitotic chromosomes were denatured with 70% formamide in 2x SSC at 85 °C for 2 minutes and dehydrated in an increasing alcohol series: 70%; 90% and 100% ethanol at -20 °C for 5 minutes each. The hybridization mixture (50% formamide, 2x SSC (Saline-sodium citrate buffer), 10% dextran sulfate, 50-100 ng labeled probe) was denatured at 95 °C for 8 min. The hybridization took place for at least 16 hours.

The 5S (pTa794) and 45S rDNA (pTa71) probes used came from the genome of *Triticum aestivum*. Probe detection was made with streptavidin antibody conjugated with alexafluor 488 and anti-digoxigenin antibody conjugated with rhodamine (Roche Diagnostics, Indianapolis, IN) after stringent washes with 2x SSC buffer and TNT (Tri-HCl, NaCl and Tween 20). Chromosomes were counterstained with DAPI in antifade mounting medium VectorShield (Vector Laboratories, Burlingame, CA). Images were captured using a CCD (charge-coupled device) camera (Retiga EXi QImaging) coupled to an Olympus BX60 fluorescence microscope and the final contrast made with Photoshop CS5.

At least 10 metaphases were evaluated for each species/cultivar and data on chromosomal morphology were obtained from the five best which presented similar level of chromosome condensation in each species. Measurements were taken with the software Micro Measure (Colorado State University). The parameters used for karyotypic studies were CL (total chromosome length – µm); LA (long arm length – µm); SA (short arm length – µm); RL (relative length – %); AR (arm ratio) and TLHS (total length of the haploid set – µm). The nomenclatures used to describe the chromosome morphology and rDNA position are proposed by Levan et al. (1964) and Roa and Guerra (2012), respectively. The karyotype classification was based on the categories established by Stebbins (1958) and intrachromosomal (A1) and interchromosomal (A2) asymmetry indices were calculated following Zarco (1986).

## Results

**Figure 1.**
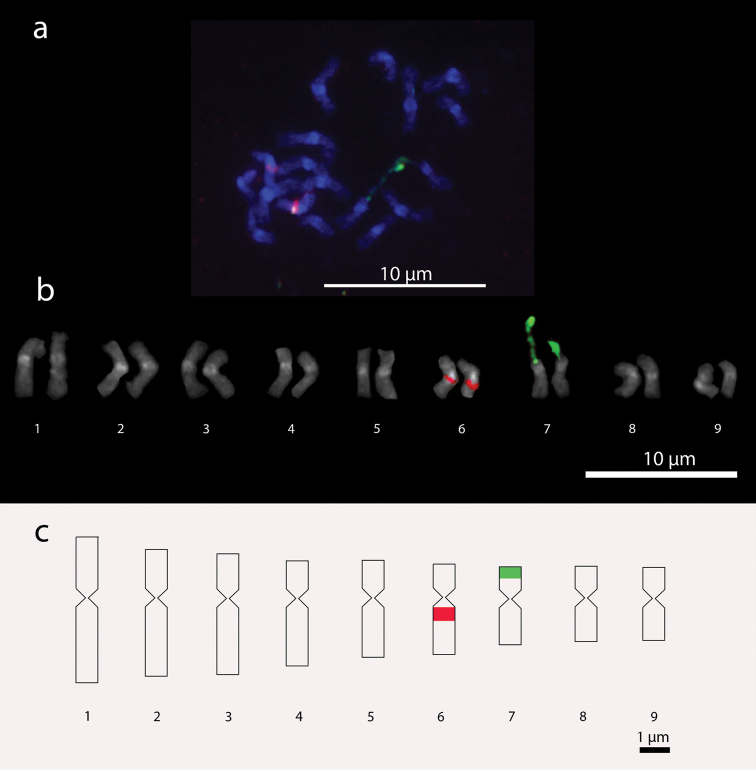
*Setaria italica* chromosomes. **a** Metaphase with 2n=18 chromosomes **b** Karyogram **c** Idiogram. In green, 45S rDNA signals; in red, 5S rDNA signals.

**Figure 2.**
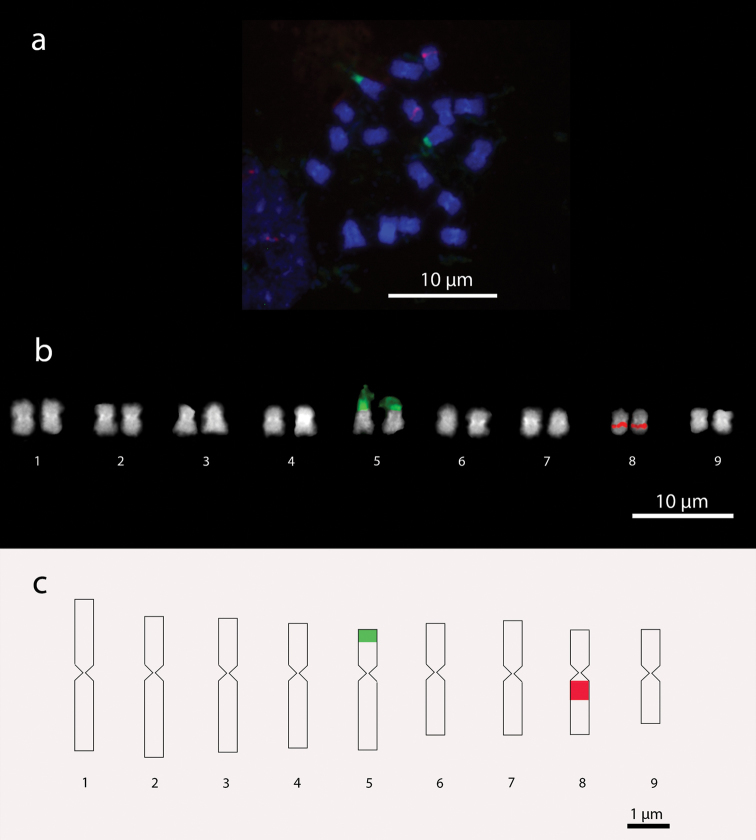
*Setaria viridis* chromosomes. **a** Metaphase with 2n=18 chromosomes **b** Karyogram **c** Idiogram. In green, 45S rDNA signals; in red, 5S rDNA signals.

*Setaria italica* presented 2n=18 chromosomes, with karyotype formula 6m+3sm and *Setaria viridis* presented the same chromosome number, however, the karyotype formula is 9m (Figures 1A and 2A). In *Setaria italica*, the relative length of the largest and the smallest chromosome pairs corresponds to 15.15% and 7.43%, respectively, and in *Setaria viridis*, 13.25% and 8.25%, respectively (Table 1).

**Table 1.** Morphometry of chromosomes of *Setaria viridis* and *Setaria italica*: CL (total chromosome length – µm); LA (long arm length – µm); SA (short arm length – µm); RL (relative length – %); AR (arm ratio) and TLHS (total length of the haploid set – µm). Metacentric (m) and submetacentric (sm) chromosomes according to Levan (1964).

*Setaria italica*							*Setaria viridis*						
Pair	CL	LA	SA	RL	AR	Class.	Pair	CL	LA	SA	RL	AR	Class.
1	4.89	2.83	2.06	15.15	1.37	m	1	3.52	1.84	1.68	13.25	1.10	m
2	4.21	2.60	1.61	13.04	1.61	m	2	3.27	1.98	1.29	12.31	1.53	m
3	4.06	2.59	1.47	12.58	1.76	sm	3	3.10	1.86	1.24	11.67	1.50	m
4	3.54	2.27	1.27	10.97	1.79	sm	4	2.87	1.76	1.11	10.81	1.59	m
5	3.23	1.97	1.26	10.01	1.56	m	5*	3.99	1.76	2.23	15.02	1.27	m
6	3.01	1.87	1.14	9.32	1.64	m	6	2.64	1.46	1.18	9.94	1.24	m
7*	4.15	2.63	1.52	12.86	1.73	sm	7	2.58	1.47	1.11	9.71	1.32	m
8	2.79	1.59	1.20	8.64	1.33	m	8	2.40	1.45	0.95	9.04	1.53	m
9	2.40	1.40	1.00	7.43	1.40	m	9	2.19	1.19	1.00	8.25	1.19	m
**TLHS**	32.28						**TLHS**	26.56					

* Chromosome pair with satellite showing extended chromatin.

Satellites were identified in chromosome pair 7 for *Setaria italica* and pair 5 for *Setaria viridis*, which were confirmed by chromosome hybridization with 45S rDNA probe by FISH technique. Satellites in both species exhibited extended DNA fibers with length ranging from 0.8 to 2.42 µm in *Setaria italica*, and 1.14 to 1.75 µm in *Setaria viridis*. The extent of chromatin overestimated the total length of these chromosome pairs. Signals of 45S rDNA have an average distance from the centromere of 0.54 µm for *Setaria italica* and 0.77 µm for *Setaria viridis*. The presence of two proximal-interstitial 5S rDNA signals were identified in *Setaria italica* on par 6 with average size of 0.48 µm and average distance of 0.34 µm from the centromere. In *Setaria viridis*, signals of 5S rDNA were identified in pair 8, with average sized of 0.40 µm and average distance of 0.17 µm from the centromere (Figures 1B and 1C, 2B and 2C).

**Figure 3.**
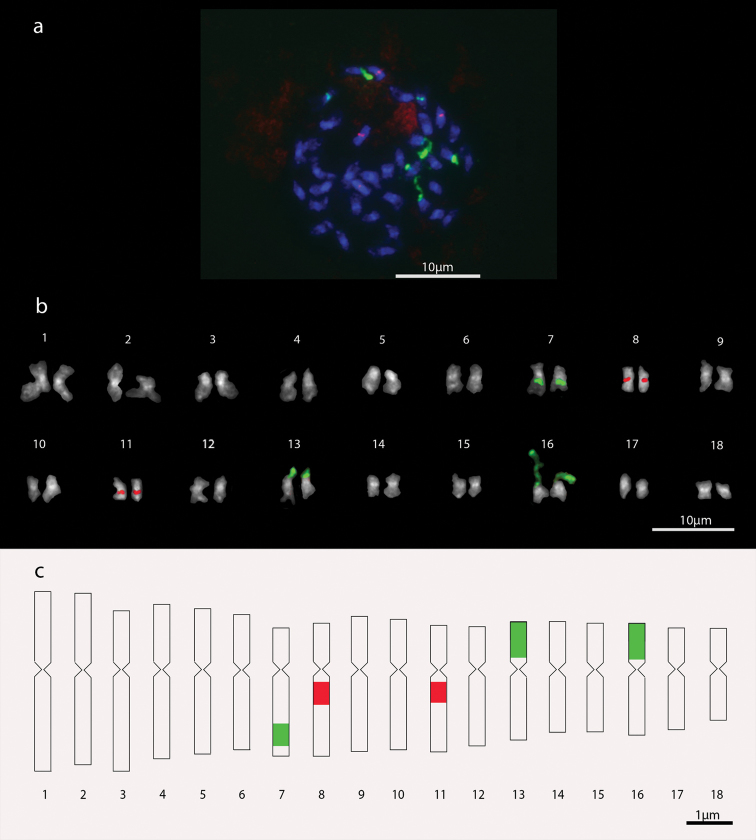
*Setaria sphacelata* cv. Narok chromosomes. **a** Metaphase with 2n=36 chromosomes **b** Karyogram **c** Idiogram. In green, 45S rDNA signals; in red, 5S rDNA signals.

**Figure 4.**
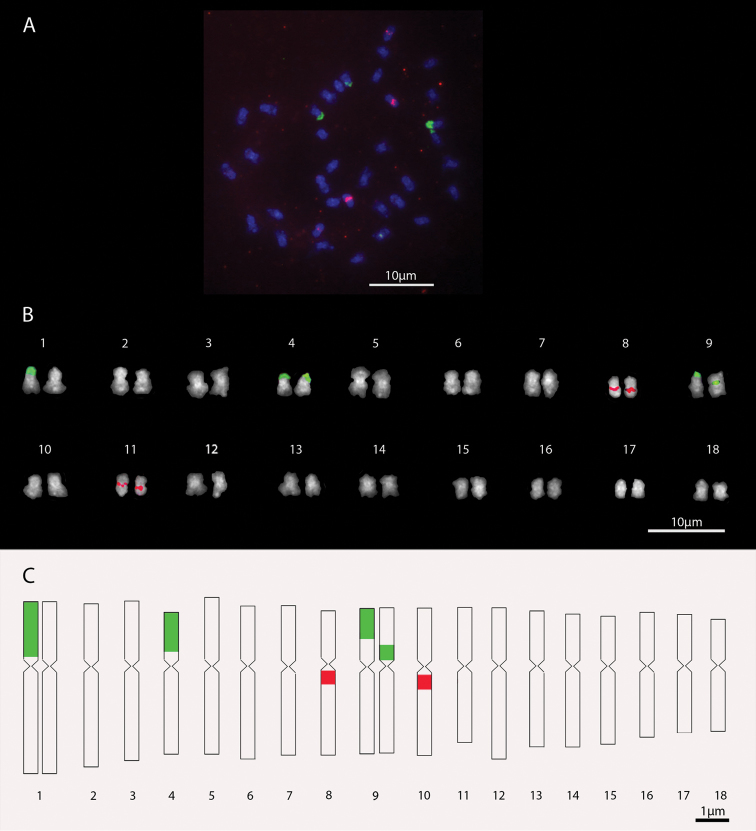
*Setaria sphacelata* cv. Nandi chromosomes. **a** Metaphase with 2n=36 chromosomes **b** Karyogram **c** Idiogram. In green, 45S rDNA signals; in red, 5S rDNA signals.

*Setaria sphacelata*, cultivars Narok and Nandi, presented 2n=36 chromosomes (Figures 3A and 4A). The cultivar Narok has karyotype formula 11m+7sm. The largest and smallest chromosome pairs have relative length of 7.03% and 3.59%, respectively (Table 2). Satellites were found in pairs 13 and 16, also confirmed by chromosome hybridization with 45S rDNA probe. The satellite region also showed extended DNA fibers with length ranging from 0.9 to 7.71 µm, with an average distance from the centromere of 0.81 and 0.66 µm for pairs 13 and 16, respectively. The FISH technique also located two interstitial-terminal 45S rDNA signals in chromosome pair 7 with average size of 0.50 µm and average distance from the centromere of 1.02 µm. The 5S rDNA signals were located at proximal-interstitial regions in chromosome pairs 8 and 11, with respective sizes of 0.49 and 0.42 µm, with average distance from the centromere of 0.22 and 0.20 µm, respectively (Figures 3B and 3C).

**Table 2.** Morphometry of chromosomes of *Setaria sphacelata* cvs. Narok and Nandi: CL (total chromosome length – µm); LA (long arm length – µm); SA (short arm length – µm); RL (relative length – %); AR (arm ratio) and TLHS (total length of the haploid set – µm). Metacentric into (m) and submetacentric (sm) chromosomes according to Levan (1964).

*Setaria sphacelata* cultivar Narok							*Setaria sphacelata* cultivar Nandi						
Pair	CL	LA	SA	RL	AR	Class.	Pair	CL	LA	SA	RL	AR	Class.
1	3.92	2.24	1.68	7.03	1.33	m	1**	2.58	1.61	0.97	6.71	1.66	m
2	3.74	2.10	1.64	6.71	1.28	m	2	2.43	1.51	0.92	6.32	1.64	m
3	3.50	2.22	1.28	6.28	1.73	sm	3	2.39	1.41	0.98	6.22	1.44	m
4	3.37	1.97	1.40	6.04	1.41	m	4***	2.33	1.43	0.9	6.06	1.59	m
5	3.16	1.86	1.30	5.67	1.43	m	5	2.31	1.31	1.00	6.01	1.31	m
6	2.98	1.79	1.19	5.34	1.50	m	6	2.3	1.39	0.91	5.98	1.53	m
7	2.95	2.05	0.90	5.29	2.28	sm	7	2.23	1.33	0.9	5.80	1.48	m
8	2.94	1.93	1.01	5.27	1.91	sm	8	2.18	1.38	0.8	5.67	1.73	sm
9	2.93	1.79	1.14	5.25	1.57	m	9****	2.16	1.31	0.85	5.62	1.54	m
10	2.87	1.77	1.10	5.15	1.61	m	10	2.15	1.32	0.83	5.59	1.59	m
11	2.77	1.80	0.97	4.97	1.86	sm	11	2.06	1.18	0.88	5.36	1.34	m
12	2.65	1.71	0.94	4.75	1.82	sm	12	2.05	1.3	0.75	5.33	1.73	sm
13*	4.85	3.32	1.53	8.70	2.17	sm	13	2.04	1.21	0.83	5.31	1.46	m
14	2.42	1.38	1.04	4.34	1.33	m	14	1.98	1.21	0.77	5.15	1.57	m
15	2.38	1.38	1.00	4.27	1.38	m	15	1.93	1.18	0.75	5.02	1.57	m
16*	4.10	2.70	1.40	7.35	1.93	sm	16	1.86	1.06	0.80	4.84	1.33	m
17	2.24	1.33	0.91	4.02	1.46	m	17	1.78	1.01	0.77	4.63	1.31	m
18	2.00	1.12	0.88	3.59	1.27	m	18	1.67	0.97	0.7	4.35	1.39	m
TLHS	55.77						TLHS	38.43					

*Chromosome pair with satellite showing extended chromatin; **Chromosome pair with satellite in hemizygous state; ***Chromosome pair with satellite; ****Heteromorphic chromosome pair for the 45S rDNA site.

*Setaria sphacelata* cultivar (cv.) Nandi presented karyotype formula 16m+2sm. The largest chromosome pair has relative length of 6.71% and the smallest,of 4.35% (Table 2). Five rDNA signals were identified for this cultivar. In chromosome pair 1, only one of the chromosomes has a “whole arm” site of 45S rDNA in the short arm, with length of 1.54 µm, at 0.06 µm far from the centromere. In pair 4, 45S rDNA signals were observed in the terminal region with an average size 0.78 µm and average distance of 0.39 µm from the centromeres. The pair 9 showed heteromorphism for the size and position of the 45S rDNA site. One of the chromosomes presented 45S rDNA signal in the terminal region with average size of 0.57 µm and average distance from the centromere of 0.57 µm. In the homologous chromosome, the signal is positioned in the proximal-interstitial region of the short arm, with an average size of 0.46 µm and average distance from the centromere of 0.03 µm. The signals of 5S rDNA were positioned in the proximal, interstitial of pairs 8 and 11, as well as in the cultivar Narok, with respective average measures of 0.43 and 0.49 µm and average distances of 0.06 and 0.05 µm from the centromeres (Figures 4B and 4C).

**Figure 5.**
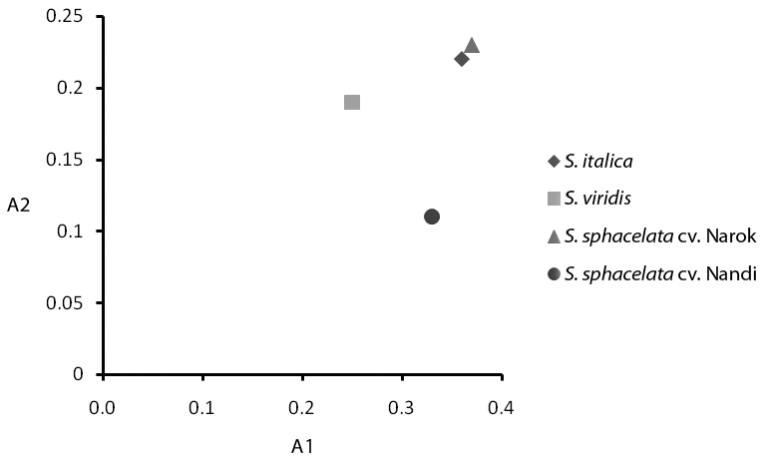
Scatter plot of karyotype asymmetry data of *Setaria* species. A1 = intrachromosomal asymmetry, A2 = interchromosomal asymmetry.

Indices of intra- (A1) and interchromosomal (A2) asymmetry showed lower values for *Setaria viridis* and *Setaria sphacelata* cv. Nandi, respectively. The highest values were found for *Setaria sphacelata* cv. Narok (Table 3). *Setaria italica* and *Setaria sphacelata* cv. Narok had closer indices (Figure 5).

**Table 3.** Chromosomal asymmetry indices in *Setaria* species, where A1 = intrachromosomal asymmetry, A2 = interchromosomal asymmetry.

	*Setaria italica*	*Setaria viridis*	*Setaria sphacelata* cv. Narok	*Setaria sphacelata* cv. Nandi
**A1**	0.36	0.25	0.37	0.33
**A2**	0.22	0.19	0.23	0.11

## Discussion

The occurrence of 2n=2x=18 chromosomes for *Setaria italica* and *Setaria viridis* corroborates the description of chromosome number previously found by different authors (Sharma and Deepesh 1956; Willweber-Kishimoto 1962; Sivaraman and Ranjekar 1984; Croullebois and Siljak-Yakovlev 1989).

The morphology of chromosomes in *Setaria italica* coincides with the findings of Croullebois and Siljak-Yakovlev (1989) for the Chinese variety Glutineux rouge, except for pair 2, in which the authors classify it as submetacentric and, in this study, the same pair is classified as metacentric. The varieties of *Setaria italica* studied by Croullebois and Siljak-Yakovlev (1989) showed differences in chromosome morphology, which were attributed to chromosomal rearrangements that have occurred during the domestication of the species, from a wild ancestor of *Setaria viridis* or two wild types already differentiated from *Setaria italica*.

In metaphases of *Setaria italica* studied, the satellites were located on chromosome pair 7. The variation found for the total length of this pair may be due to the late condensation of chromosomes in the terminal region, which was also reported by Croullebois and Siljak-Yakovlev (1989) for the species. The extent of chromatin in the satellite area may also be related to slide preparation with a tendency to promote detachment of the satellite. Croullebois and Siljak-Yakovlev (1989) also observed satellites in chromosome pair 7. The authors also verified, in some metaphases, the presence of a second pair of satellites, apparently located on chromosome pair 6 or 8. The presence of two chromosome pairs with satellite was previously only reported by Sharma and Deepesh (1956) in analysis on plants from Mumbai, India, however, the satellite was located on chromosome pairs 1 and 2. The differences observed for the satellite position can be attributed to the origin of the evaluated genotype, however, the satellite depends on activation/deactivation of the NOR. Therefore this cytogenetic marker is very plastic and variable.

The number of signals of 45S and 5S ribosomal genes found for *Setaria italica* and *Setaria viridis* coincides with previous analyses carried out by Benabdelmouna et al. (2001), however, the authors failed to identify in which pairs the signals were present.

The karyotypes of varieties of *Setaria italica* and *Setaria viridis* described herein are classified as symmetrical, according to Stebbins (1958), as also observed in varieties Glutineux rouge, Burganjou of *Setaria italica* evaluated by Croullebois and Siljak- Yakovlev (1989) using the criteria of Arano and Saito (1980).

According to the asymmetry indices patterns set by Stebbins (1958), the karyotype of *Setaria italica* was included in the category 1B and *Setaria viridis* in the category 1A. The species were included in different categories since *Setaria italica* have some of the chromosomes classified as submetacentric and because *Setaria viridis* has a higher ratio between the largest and smallest chromosomes, justifying the divergence of these species in relation to the asymmetry indices proposed by Zarco (1986). According to Stebbins (1958), less asymmetric karyotypes are characterized by the predominance of metacentric chromosomes of similar size, typical of species with less specialized and phylogenetically more basal karyotypes. Considering the criteria proposed by Zarco (1986), *Setaria italica* and *Setaria viridis* do not have the same tendencies of intra- and interchromosomal asymmetry and *Setaria italica* was closer to the tetraploid *Setaria sphacelata* cv. Narok.

*Setaria italica* is a species that was domesticated from *Setaria viridis* in northern China around 6000 BC (Bettinger et al. 2010). These species are apparently very similar and there are no enough characters for taxonomic separation (Kawase and Sakamoto 1984; Wang et al. 1998; Benabdelmouna et al. 2001). The morphological and chromosomal differences probably occurred due to domestication (Harlan and De Wet 1971; Benandelmouna et al. 2001). The presence of all metacentric chromosomes in *Setaria viridis*, a typical characteristic of basal species, reinforces the hypothesis of ancestry relationship with *Setaria italica*.

The analysis on karyotype asymmetry, the classification of chromosome pairs 3, 4 and 7 as submetacentric in *Setaria italica*, the relative length of the largest and the smallest chromosomes different between *Setaria italica* and *Setaria viridis* and differences in the position and size of 45S and 5S rDNA signals indicate chromosomal rearrangements and/or amplifications in the diversification process between these species.

Sequence data of the genome of *Setaria italica* and *Setaria viridis* done by Bennetzen et al. (2012) showed that transposable elements are abundant, newly activated and non-randomly distributed in the genome of these species. Because of the nature of promoting breaks in chromosomes, acquiring and amplifying genes or fragments of genes and serving as recombination sites (Bennetzen 2005), the transposable elements are likely candidates for participation in macro and micro chromosomal rearrangements (Bennetzen et al. 2012).

Cultivars of *Setaria sphacelata* presented 2n=4x=36 chromosomes. According to Hacker and Jones (1969), the species has basic chromosome number of x=9, but presents cultivars and varieties with chromosome numbers ranging from 2n=18 to 2n=90.

Details of chromosome morphology for *Setaria sphacelata* are first described in this paper. Differences between cultivars were found with respect to the size of the largest and smallest chromosome pairs. By comparison, the chromosome pair 3, 7, 11, 13 and 16 differ in relation to the centromere position, while the others were similar. The chromosome pairs 8 and 11, carrying the 5S rDNA signals, are preserved, but differed in the position of the centromere. The 45S rDNA signals also showed variation in size, probably by means of amplification and/or rearrangements, in addition to late condensation of the terminal region of chromosomes.

*Setaria sphacelata* cv. Narok and Nandi have symmetrical karyotypes included in categories 2A and 1A of Stebbins (1958), respectively. The cultivar Narok has higher asymmetry rates than those of Nandi, given the presence of a greater number of chromosomes classified as submetacentric, besides having a higher ratio between the largest and smallest chromosomes. According to Stebbins (1971), increased karyotype asymmetry is due to changes in centromere position and the relative size of the chromosomes. In this way, the karyotype of *Setaria sphacelata* cv. Narok is considered more asymmetric than that of *Setaria sphacelata* cv. Nandi.

A higher number of rearrangements is usually attributed to species with more specialized karyotypes (Stebbins 1971). Nevertheless, the cultivar Nandi, despite presenting a higher number of metacentric chromosomes, also showed a chromosome pair in a hemizigous state and other heteromorphic pair for 45S rDNA signals indicating rearrangements.

Moreover, in chromosome pair 1, it is possible that one of the homologous chromosome sites has been eliminated after polyploidization. In agreement with Roa and Guerra (2012), in general, recent polyploidization events result in duplication of number of sites, but in comparisons between diploid and polyploid plants of the same genus, there is a clear trend in reducing the number of sites, leading to diploidization. Thus, chromosomal duplication makes the number of copies genes greater than necessary, and the loss of some repeated sequences causes no deleterious effects on plant species (Phillips 1978, Rogers and Bendich 1987, Winterfeld and Roser 2007).

Inactive sites of 45S rDNA are more likely to be polymorphic and eventually be eliminated. The dynamic inactivation and subsequent deletion seems to neutralize the duplication and dispersion of repeated ribosomal genes, leading to the observation of a lower number of sites in the species (Roa and Guerra 2012).

The heteromorphic state found in par 9 for the 45S rDNA signal in *Setaria sphacelata* cv. Nandi may be due to different events, such as intrachromosomal translocation, transposable elements and inversions. Ribosomal DNA has high potential for intragenomic mobility causing chromosome polymorphisms (Schubert and Wobus 1985). Blocks of ribosomal genes can suddenly change their position without any other change in the other remaining chromosomal marks (Dubcovsky and Dvorak 1995). Nucleolus organizer regions in *Allium* Linnaeus, 1753 species can jump in the genome to apparently non-random sites, however, it is still unclear whether the unequal recombination or transposition are the mechanisms responsible for the mobility of these sites (Schubert and Wobus 1985; Schubert 2007).

The occurrence of chromosomes in hemizygous has already been reported for other grasses, such as *Lolium* Linnaeus, 1753 (Rocha et al. 2015), *Paspalum* Linnaeus, 1759 (Vaio et al. 2005) and *Hordeum* Linnaeus, 1753 (Taketa et al. 2001). The mobility of genes, polymorphism in the original population, reduction in the site size and the deletion of genes are mechanisms for karyotypic evolution that may be involved in the origin of heteromorphism of the rDNA locus (Vaio et al. 2001).

The mobility of rDNA sites caused by transposons has already been confirmed in wheat. EN/Spm transposable elements, for example, have the ability to capture entire genes and to move them to different parts of the genome (Jiang et al. 2004; Lai et al. 2005), furthermore can form clusters, associated or not with rDNA regions, which weaken the chromosome structure causing breakage and subsequent karyotypic remodeling (Raskina et al. 2004).

Another hypothesis for the polymorphism in chromosome pair 9 can be the paracentric inversion in the short arm of one of the homologous chromosomes. The change in NOR position associated with inversion is described by Schubert (2007) as an important rearrangement for karyotype evolution, and had already been reported for *Arabidopsis thaliana* (Linnaeus, 1753) Heynhold, 1842 (Lysak et al. 2006), and hybrids between *Avena insularis* Ladizinsky, 1998 and *Avena murphyi* Ladizinsky, 1971 (Ladizinsky 1999). Confirmation of inversion and analysis of consequences in *Setaria sphacelata* cv. Nandi should be performed with analysis in meiotic cells at pachytene.

## Conclusions

The number and position of the 5S rDNA sites are stable for the species studied.

There is intraspecific and interspecific variation for the number and location of 45S rDNA sites in *Setaria*.

*Setaria sphacelata* cultivars can be distinguished by means of karyotype analysis, which revealed chromosomal rearrangements in the evolutionary process.
